# Tympanitis Cured with Carbolic Acid

**Published:** 1890-07

**Authors:** Claude D. Morris


### TYMPANITIS CURED WITH CARBOLIC ACID.

By Claude D. Morris, V.S.

March 18th, I was called to see a Holstein yearling bull, suffering with tympanitis. When I arrived at the yard the animal in question was bloated so badly that I at once used the trocar; relief was speedy. Upon inquiry I found that the animal had been recently purchased, and the present owner was endeavoring to grow him rapidly. Previously he had been kept on a moderate allowance of good food, but at this time was receiving all the good clover hay he would eat and a liberal allowance of corn. Upon examination I found that there was slight impaction of the rumen and omasum. I administered sulphate of magnesia one and one-half pounds, aromatic spirits of ammonia one ounce. Left the animal with instructions, and to report the next morning the condition of the animal, and especially regarding the bowels. At the stated time the care-tender came to my office and said that the bowels had moved freely, but the bloating had returned after giving him his gruel. I ordered the ammonia repeated, and to alternate it with colocynth every hour so long as the bloating continued. This, however, proved fruitless, and the strange part of it all is, the animal continued to bloat every day for three weeks, the only relief being the use of the hollow probang. For three weeks I tried every reasonable means to overcome this trouble, and finally resorted to carbolic acid ♏xx in water q. s. twice each day for one week. The bloating did not return after the first dose of carbolic acid. During the period of gastric trouble the animal partook anxiously of food when offered, it being milk, gruel and a small allowance of hay, the bowels also remaining normal.

